# Multiple craniotomies in patients with brain metastases: a two-center, propensity score-matched study

**DOI:** 10.1007/s10143-024-02578-8

**Published:** 2024-07-26

**Authors:** Luis Padevit, Anna Maria Zeitlberger, Nicolai Maldaner, Johannes Sarnthein, Oliver Bozinov, Luca Regli, Marian Christoph Neidert, Carlo Serra, Stefanos Voglis

**Affiliations:** 1 Department of Neurosurgery, Clinical Neuroscience Center, University Hospital and University of Zurich, Rämistrasse 100, Zurich, 8091 Switzerland; 2 Department of Neurosurgery, Cantonal Hospital St. Gallen, St. Gallen, Switzerland

**Keywords:** Brain metastases, Adverse events, Complications, Surgical resection, Targeted therapy, Immunotherapy

## Abstract

The current study addresses the question of whether the resection of more than one BM by multiple craniotomies within the same operation is associated with more adverse events (AEs) and worse functional outcome compared to cases in which only one BM was resected. All patients who underwent more than one craniotomy for resection of multiple BM at two Swiss tertiary neurosurgical care centers were included. Any AEs, functional outcome, and overall survival (OS) were analyzed after 1:1 propensity score matching with patients who underwent removal of a single BM only. A total of 94 patients were included in the final study cohort (47 of whom underwent multiple craniotomies). There was no significant difference in the incidence of AEs between the single and the multiple craniotomy group (*n* = 2 (4.3%) vs. *n* = 4 (8.5%), *p* = .7). Change in modified Rankin Scale (mRS) and Karnofsky Performance Status (KPS) at discharge demonstrated that slightly more single craniotomy patients improved in mRS, while the proportion of patients who worsened in mRS (16.3 vs. 16.7%) and KPS (13.6 vs. 15.2%) was similar in both groups (*p* = .42 for mRS and *p* = .92 for KPS). Survival analysis showed no significant differences in OS between patients with single and multiple craniotomies (*p* = .18). Resection of multiple BM with more than one craniotomy may be considered a safe option without increased AEs or worse functional outcome.

**Supplementary Information:**

The online version contains supplementary material available at 10.1007/s10143-024-02578-8.

## Introduction

Brain metastases (BM) continue to pose a significant challenge in oncology, representing the most common central nervous system tumor. Recent studies have suggested new targeted as well as immunotherapeutic treatment options [1]. Microsurgical resection and stereotactic radiosurgery however, remain the cornerstone of local control [2], particularly in solitary lesions, where it has demonstrated safety and efficacy even in deep-seated lesions [3]. When faced with multiple BM, clinicians must make the critical decision of whether to proceed immediately with therapies such as radiation or chemotherapy or, in selected cases, consider microsurgical resection of multiple lesions. This dilemma becomes particularly relevant in cases of large and symptomatic metastases, but also in cases of small lesions with significant edema, where surgical intervention through multiple craniotomies may be warranted to relieve intracranial pressure or reduce perilesional edema to facilitate the initiation of adjuvant therapy.

Despite the clinical importance of this issue, the existing evidence on structured outcome and complications in patients with multiple craniotomies for BM remains limited. To address this gap, we present a modern microsurgical propensity score-matched cohort analysis conducted at two Swiss tertiary neurosurgical referral centers focusing on the rates of adverse events (AEs), surgical morbidity, and mortality associated with multiple craniotomies for patients with multiple BM. We aim to provide valuable insights into the safety and feasibility of this surgical approach in the management of patients with multiple BM.

## Materials & methods

### Study cohort, data acquisition and ethical considerations

All patients who underwent resection of at least two distinct and histologically confirmed BM through more than one craniotomy within the same surgery between January 2012 and June 2022 at the Department of Neurosurgery, University Hospital Zurich (USZ) and between January 2014 and August 2022 at the Department of Neurosurgery, Cantonal Hospital St. Gallen (KSSG) were considered for propensity score matching. The primary outcome was defined as the rate of AEs at discharge and the main secondary outcome, the functional outcome at discharge, was measured by the Modified Rankin scale (mRS) and Karnofsky Performance Scale (KPS). Patient records of USZ patients were extracted from a prospectively recorded institutional registry [4]. All procedures performed in studies involving human participants were in accordance with the Ethical Standards of the Institutional and/or National Research Committee and with the 1964 Helsinki Declaration and its later amendments or comparable ethical standards. This article does not report animal studies. The registry was approved upfront by the local ethics review board (PB-2017-00093) and internationally registered at clinicaltrials.gov (NCT01628406). Patient data from KSSG were extracted from a retrospective registry including all craniotomies performed for brain tumors (approved by the local ethics review board, identifier: 2023 − 01343). Parameters extracted included patient demographics, lesion, surgical and outcome characteristics. mRS and KPS were used as general clinical performance scales. All surgeries were performed or closely supervised by experienced attending neurosurgeons, intraoperative neuronavigation was used as a standard for all cases. Complications were defined as any deviation of the expected postoperative course and are graded according to the Clavien-Dindo grading system (CDG, see online resource 1) [5] or the Therapy-Disability-Neurology grade (TDN, see online resource 2) [6]. For each case only the complication with the highest CDG at discharge entered further analysis. Anatomic lobe and gyral location of resected BM were additionally extracted. Pre - and postoperative T1-weighted MRI images with gadolinium contrast were used for anatomical localization of the BM and were confirmed by an experienced consultant neurosurgeon and the corresponding neuroradiological reports.

### Statistical analysis

All data processing and analysis steps were performed with R Studio (Version 2023.06.0, R Studio Inc. and R version 4.3.0) [7] using open-source libraries.

A 1:1 nearest neighbor propensity score matching without replacement was performed using the *Matching* R package [8]. Covariate balance was assessed using the lovePlot function of the *cobalt* R package [9]. Matching results showed that the standardized mean differences for all covariates were below the set cutoff of 0.1 (see online resource 3) indicating an adequate balance. Matching used all 47 “treated” cases (patients with multiple craniotomies) and no patients were discarded during the matching process.

Plotting of anatomical locations on a reference brain atlas was done using the *coldcuts* R package [10] and corresponding craniotomy/lesion localization using the *circlize* R package [11]. Missing values were considered missing at random and therefore omitted from the respective analysis. Continuous variables are given as means and standard deviation (SD) whereas categorical variables are reported as numbers and percentages of total. All statistical tests used are indicated in the figure captions or the main text. P-values < 0.05 were considered statistically significant. Anonymized raw data and analysis scripts are available from the corresponding author upon reasonable request.

## Results

### Study cohort characteristics

Of 60 patients that underwent multiple craniotomies, 47 (31 USZ and 16 from KSSG cohort) were included into the study, while the remainder was excluded due to missing data in the variables used for matching. After propensity score matching, the final study cohort thus consisted of 94 patients. Matching was performed for age (cutoff ≥ 70 years), sex, preoperative KPS, primary surgery, American Society of Anesthesiologists (ASA) risk classification as well as infratentorial and perirolandic location/involvement of the BM (Table 1 shows those parameters for the two subgroups and indicates adequate matching). Mean age of the study cohort was 57.6 years (56.6 in the single craniotomy vs. 58.7 years in the multiple craniotomies subgroup). 57% of patients were male and 87% cases were primary surgeries, whereas the rest underwent previous BM resection. 67% of cases had metastases located supratentorial and only 6% perirolandic. See online resource 4 for anatomical distribution of all study cohort patients and corresponding BM locations for the multiple craniotomies group. In the multiple craniotomies group, 92% of patients had 2 and 8% 3 craniotomies. As expected, mean surgery time differed significantly between the single and multiple craniotomies group (213 vs. 271 min, *p* = .004). Looking at the entire study cohort most common primary tumors were lung carcinomas (43%, *n* = 40), followed by melanoma (20%, *n* = 19) and breast cancer (16%, *n* = 15) as depicted in Table 2. In single craniotomy patients 51% had single metastases, followed 2–4 metastases in 21% (vs. 77% in multiple craniotomies patients), 5–10 in 15% (9.3%) and > 10 in 13% (vs. 14%, *p* < .001).

**Table 1:** Balanced demographic and baseline characteristics

Characteristic	Overall, *N* = 94^*1*^	Craniotomies		*p*-value^2^
		Single, *N* = 47^*1*^	Multiple, *N* = 47^*1*^	
Age category*				0.8
<70	73 (77.7%)	37 (78.7%)	36 (76.6%)	
≥70	21 (22.3%)	10 (21.3%)	11 (23.4%)	
**Age**	57.6 (14.8)	56.6 (16.0)	58.7 (13.6)	0.7
Sex*				> 0.9
Male	54 (57.4%)	27 (57.4%)	27 (57.4%)	
Female	40 (42.6%)	20 (42.6%)	20 (42.6%)	
KPS preoperative*				0.8
≥70	67 (71.3%)	33 (70.2%)	34 (72.3%)	
<70	27 (28.7%)	14 (29.8%)	13 (27.7%)	
Primary surgery*				> 0.9
Yes	82 (87.2%)	41 (87.2%)	41 (87.2%)	
No	12 (12.8%)	6 (12.8%)	6 (12.8%)	
ASA risk classification*				> 0.9
3	51 (54.3%)	26 (55.3%)	25 (53.2%)	
2	35 (37.2%)	17 (36.2%)	18 (38.3%)	
4	8 (8.5%)	4 (8.5%)	4 (8.5%)	
Infratentorial location*				0.8
No	63 (67.0%)	32 (68.1%)	31 (66.0%)	
Yes	31 (33.0%)	15 (31.9%)	16 (34.0%)	
Perirolandic location*				> 0.9
No	88 (93.6%)	44 (93.6%)	44 (93.6%)	
Yes	6 (6.4%)	3 (6.4%)	3 (6.4%)	

Statistics presented: n (%); Mean (SD). Pearson’s Chi-squared test (for all *n* ≥ 5) and Fisher’s exact test (for all *n* < 5) for categorical data. Wilcoxon rank sum test for continuous data.

KPS Karnofsky Performance Status, ASA American Society of Anesthesiologists.

* Variables used for propensity score-matching.

**Table 2:** Unbalanced surgical and lesional covariables

Characteristic	Overall, *N* = 94^*1*^	Craniotomies		*p*-value^2^
		Single, *N* = 47^*1*^	Multiple, *N* = 47^*1*^	
**No. of craniotomies**				< 0.001
1	47 (50.0%)	47 (100.0%)	0 (0.0%)	
2	43 (45.7%)	0 (0.0%)	43 (91.5%)	
3	4 (4.3%)	0 (0.0%)	4 (8.5%)	
**Urgency of the operation**				0.2
Elective	67 (87.0%)	42 (91.3%)	25 (80.6%)	
Emergency	10 (13.0%)	4 (8.7%)	6 (19.4%)	
Unknown	17	1	16	
**Length of surgery [minutes]**	244.5 (98.2)	213.1 (93.1)	271.9 (95.1)	**0.004**
**Primary tumor**				0.4
Lung	40 (42.6%)	17 (36.2%)	23 (48.9%)	
Melanoma	19 (20.2%)	8 (17.0%)	11 (23.4%)	
Breast	15 (16.0%)	8 (17.0%)	7 (14.9%)	
Other	8 (8.5%)	6 (12.8%)	2 (4.3%)	
GIT	7 (7.4%)	5 (10.6%)	2 (4.3%)	
Kidney	5 (5.3%)	3 (6.4%)	2 (4.3%)	
**Number of metastases**				**< 0.001**
1	24 (26.7%)	24 (51.1%)	0 (0.0%)	
2–4	43 (47.8%)	10 (21.3%)	33 (76.7%)	
5–10	11 (12.2%)	7 (14.9%)	4 (9.3%)	
>10	12 (13.3%)	6 (12.8%)	6 (14.0%)	
Unknown	4	0	4	

Statistics presented: n (%); Mean (SD). Pearson’s Chi-squared test (for all *n* ≥ 5) and Fisher’s exact test (for all *n* < 5) for categorical data. Wilcoxon rank sum test for continuous data.

### Frequency of adverse events and clinical outcomes

The occurrence of any AE at discharge was recorded in 6.4% of the whole study cohort (*n* = 6, see Table 3). There was no significant difference between the two subgroups (*n* = 2 for the single vs. *n* = 4 for the multiple craniotomies group, *p* = .7). Two patients showed a new neurological deficit after surgery (1 in each group), 1 patient in the multiple craniotomies group a postoperative hemorrhage, corneal abrasion, and pneumonia respectively and 1 patient died within 30 days related to the surgery/postoperative course (single craniotomy group). See Table 3 for the respective CDG scores. Similarly, there was no significant difference in the severity of TDN grades between the two groups (*p* = 7, see Table 3). Length of stay for the whole study cohort was 9.2 days with no significant difference between the single and multiple craniotomy group (9.6 vs. 8.9 days respectively, *p* = .3).

**Fig. 1:**
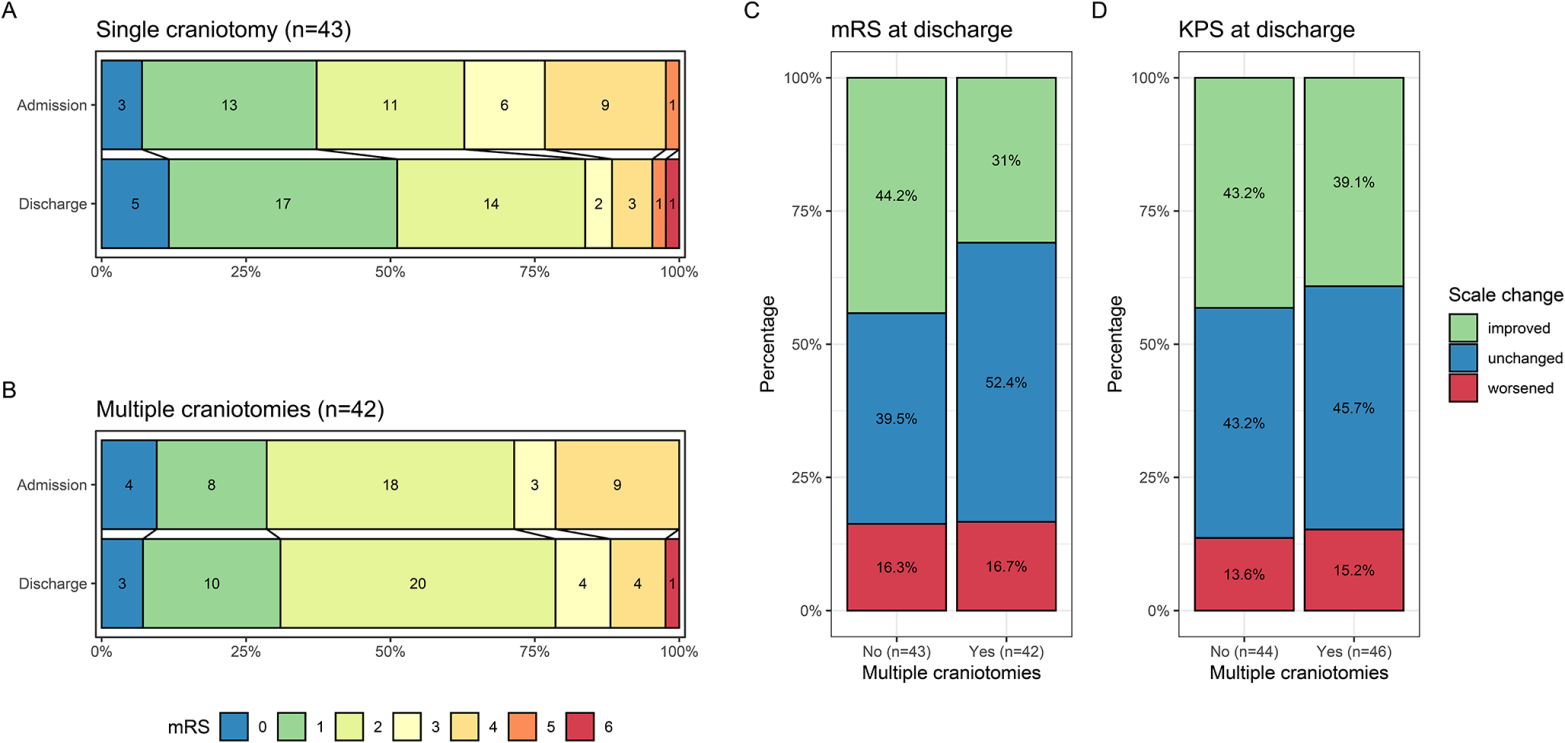
Clinical outcomes at discharge. (**A** & **B**) Percentages of mRS scores at admission (upper row) and discharge (lower row) for patients with single craniotomy (**A**) and for patients with multiple craniotomies (**B**). Changes of mRS (**C**) and KPS (**D**) at discharge relative to admission stratified for multiple craniotomies. mRS Modified Rankin Scale; KPS Karnofsky Performance Status

Figure 1 shows the clinical outcome scale changes upon discharge for the two groups in the study cohort: Patients who underwent multiple craniotomies did not have significantly higher mRS scales at discharge compared to admission (see Fig. 1B) compared to patients who did not experience any AE (see Fig. 1A). Additionally, looking at the relative changes in mRS and KPS between discharge and admission, slightly more patients with single craniotomy improved in mRS, whereas the proportion of patients worsening in mRS (16.3 vs. 16.7%, see Fig. 1C) and KPS (13.6 vs. 15.2%, see Fig. 1D) were comparable in both groups (*p* = .42 for mRS and *p* = .92 for KPS, Pearson’s chi-squared test).

**Table 3:** AE characteristics at discharge

Characteristic	Overall, *N* = 94^*1*^	Craniotomies		*p*-value^2^
		Single, *N* = 47^*1*^	Multiple, *N* = 47^*1*^	
**AE at discharge**				0.7
No	88.0 (93.6%)	45.0 (95.7%)	43.0 (91.5%)	
Yes	6.0 (6.4%)	2.0 (4.3%)	4.0 (8.5%)	
**Worst CDG at discharge**				0.6
None	88.0 (93.6%)	45.0 (95.7%)	43.0 (91.5%)	
1	2.0 (2.1%)	1.0 (2.1%)	1.0 (2.1%)	
2	2.0 (2.1%)	0.0 (0.0%)	2.0 (4.3%)	
3b	1.0 (1.1%)	0.0 (0.0%)	1.0 (2.1%)	
5	1.0 (1.1%)	1.0 (2.1%)	0.0 (0.0%)	
**Which AE at discharge**				0.8
None	88.0 (93.6%)	45.0 (95.7%)	43.0 (91.5%)	
New neurological deficit	2.0 (2.1%)	1.0 (2.1%)	1.0 (2.1%)	
Cornea erosion	1.0 (1.1%)	0.0 (0.0%)	1.0 (2.1%)	
Hemorrhage	1.0 (1.1%)	0.0 (0.0%)	1.0 (2.1%)	
Pneumonia	1.0 (1.1%)	0.0 (0.0%)	1.0 (2.1%)	
Death within 30 days	1.0 (1.1%)	1.0 (2.1%)	0.0 (0.0%)	
**TDN at discharge**				0.7
no AE	87.0 (92.6%)	45.0 (95.7%)	42.0 (89.4%)	
2	4.0 (4.3%)	1.0 (2.1%)	3.0 (6.4%)	
5	2.0 (2.1%)	1.0 (2.1%)	1.0 (2.1%)	
3	1.0 (1.1%)	0.0 (0.0%)	1.0 (2.1%)	
**Length of stay** [days]	9.2 (5.9)	9.6 (5.4)	8.9 (6.5)	0.3

Statistics presented: n (%), Fisher’s exact test.

AE adverse events, CDG Clavien-Dindo grading, TDN Therapy-Disability-Neurology grade.

### Survival analysis

**Fig. 2:**
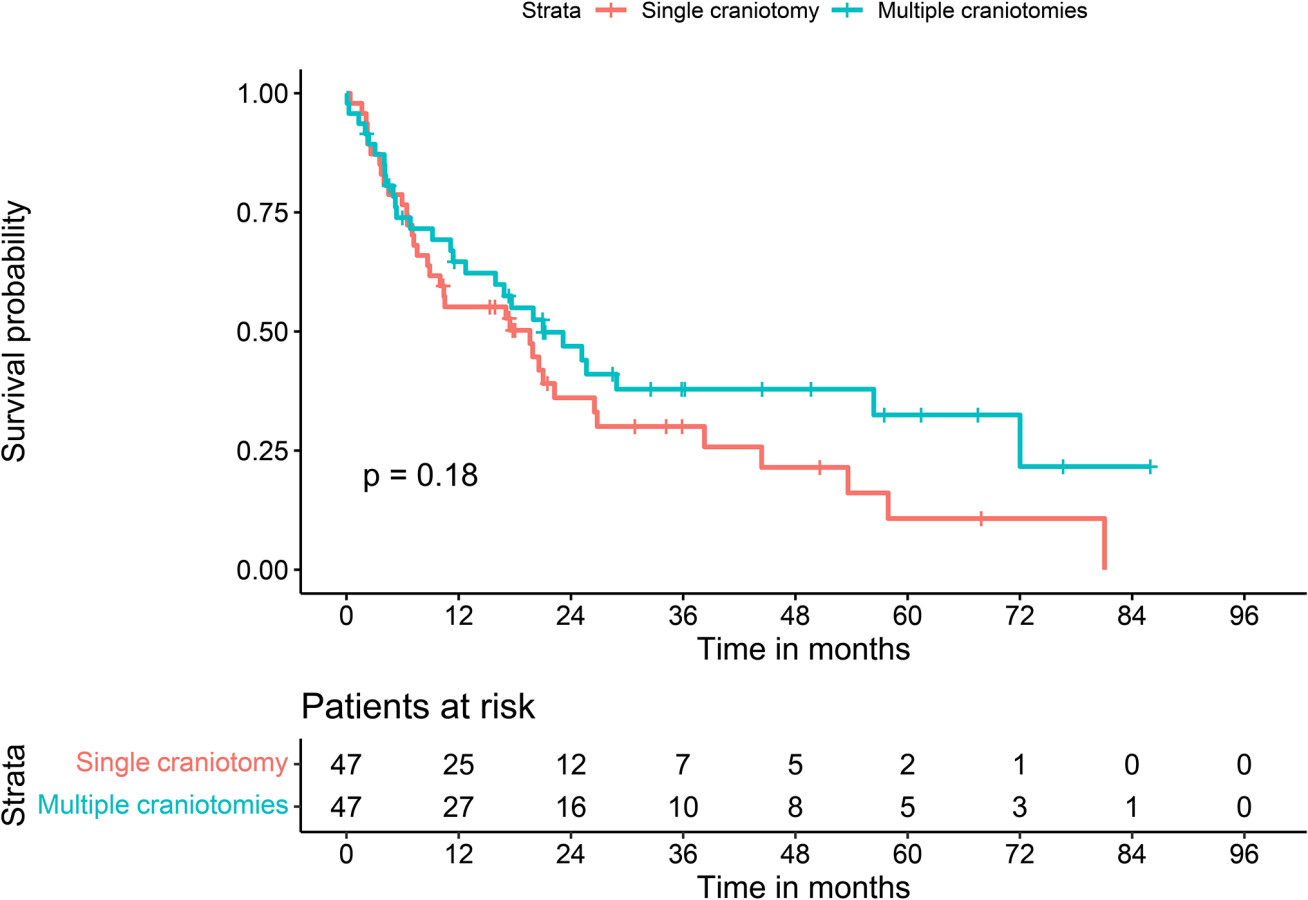
Overall survival stratified by multiple craniotomies

**Fig. 3:**
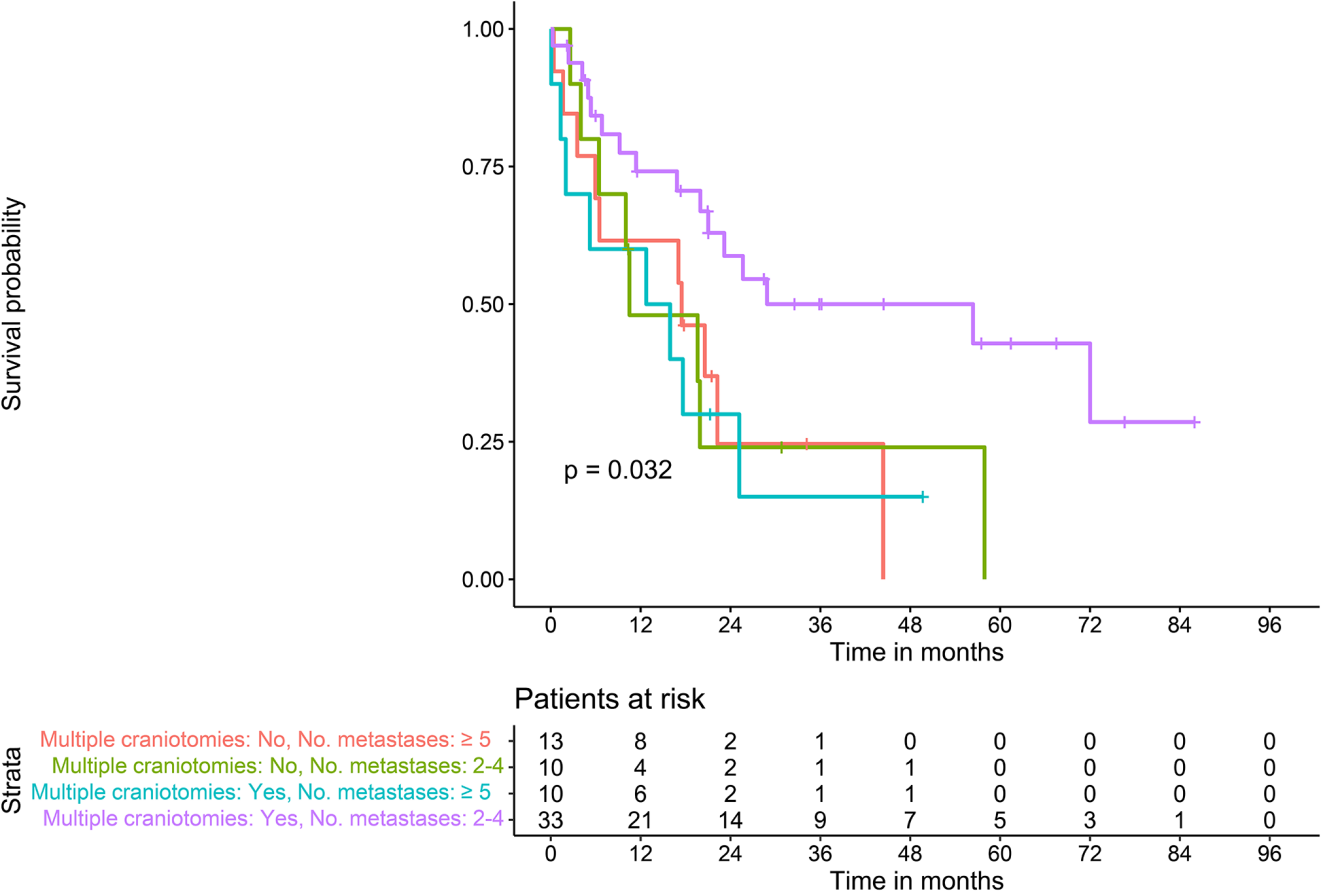
Overall survival stratified by multiple craniotomies and number of metastases for patients with multiple BMs

Looking at overall survival (OS), Kaplan-Meier analysis showed no significant differences in OS between patients with single and those with multiple craniotomies (*p* = .18, log-rank test, see Fig. 2). For all patients with more than one BM (*n* = 66), further stratification by number of BMs2-4 vs. ≥5 showed that patients with 2–4 BMs who underwent multiple craniotomies had significantly longer OS (*p* = .032, log-rank test, see Fig. 3). Interestingly the subgroup with ≥ 5 lesions and multiple craniotomies did not show any survival benefit compared to the corresponding groups without multiple craniotomies.

Kaplan-Meier curve with log-rank statistic of patients stratified by single craniotomy vs. multiple craniotomies.

Kaplan-Meier curve with log-rank statistic of patients stratified by single craniotomy vs. multiple craniotomies and number of metastases.

## Discussion

In the current study, we were able to show that resection of multiple BM through multiple craniotomies in a single operation is not associated with more or more severe AEs compared to single craniotomy operations. The removal of more than one BM in a single surgical session can thus be considered a safe therapeutic option in selected patients.

Modern multidisciplinary treatment approaches for patients with BM have changed significantly in recent years with the introduction and proven efficacy of targeted and immunotherapeutic agents [1, 12–14]. Along with stereotactic radiosurgery, microsurgical resection has traditionally been a mainstay of local control, foremost in patients with large and/or symptomatic BM. With the introduction of new techniques and tools, its safety and applicability has evolved over time and has been shown to be associated with low morbidity and mortality even in deep-seated metastatic lesions [3]. However, resection of more than one lesion in one surgical session in patients with disseminated BM is still rarely performed and surgery may be indicated in selected cases [2]: For example, in patients with few, solitary lesions to reduce intracranial tumor burden, which may be associated with better survival in some primary tumor entities [2, 15, 16], in lesions with perifocal edema to allow for faster steroid tapering for subsequent immunotherapy [1, 2] or to relieve intracranial pressure in case of occlusive hydrocephalus.

To date there has only been a limited number of studies [17–23] addressing the question of feasibility and safety of multiple craniotomies in one surgical session in BM patients. Early retrospective cohort studies or case reports such as Bindal et al. in 1993 [24] and Paek et al. in 2005 [19] showed that patients who underwent multiple craniotomies for BM had similar survival rates as well as functional outcome [19] compared to patients who underwent resection of a solitary BM. Similarly, and more recently, Baker et al. in 2017 [20] concluded that removing several BM using multiple craniotomies in one surgery is safe and feasible, while Tanei et al. in 2019 [22] described 3 cases where more than one BM was removed in one surgery, although one case was an atypical meningioma. All patients significantly increased in terms of functional outcome post-surgery, information about possible AEs was however not provided.

With this study, we present the largest available series of patients undergoing multiple craniotomies for disseminated BM. Our analysis demonstrates that multiple craniotomies within a single surgery are not necessarily associated with increased morbidity and perioperative AEs and have a similar functional outcome in terms of mRS and KPS. Our results are in line with a recent report by Bschorer et al. which showed in a series of 30 patients with 2–3 craniotomies per patient, that multiple craniotomies were not associated with increased perioperative complications, a worse functional or neurological outcome [18]. Removal of multiple BM in a single surgical session may therefore be a valid therapeutic option in selected patients with a low-risk profile.

As the overall evidence of a positive prognostic effect on oncological and survival outcomes of minimizing residual intracerebral tumor volume is limited, further studies are needed to demonstrate that resection of multiple disseminated BM within a patient has a real benefit in terms of overall/progression-free survival. Our study shows that performing multiple craniotomies in one surgical session would not necessarily be associated with an increase of perioperative AEs.

## Limitations

Due to the retrospective design of the study, all its afflicted limitations apply to this study as well. Regarding the anatomical localization of the lesions, inter-surgeon variability is also a possible source of bias. However, to minimize this, each anatomical localization was reconfirmed by the authors of this study. In addition, although propensity score matching was used to generate an appropriate comparison group, due to the limited number of cases, matching variables were focused only on parameters relevant to AE occurrence, as this was the focus of the current study. To thoroughly address questions regarding oncologic and survival outcomes, larger cohorts that include primary tumor and oncologic treatment parameters in the matching process or, ideally, prospective studies are needed.

## Conclusion

In individual, oncologically carefully selected patient cases, removal of more than one BM using multiple craniotomies in one surgical session seems to be a safe and valid option with a low risk of AEs and good functional outcome.

## Electronic supplementary material

Below is the link to the electronic supplementary material.

Supplementary Material 1

## Data Availability

The datasets generated during and/or analysed during the current study are available from the corresponding author on reasonable request.
