# Supplementary material for: Multiple craniotomies in patients with brain metastases: a two-center, propensity score-matched study
Source: Neurosurg Rev. 2024 Jul 26;47(1):354. doi: 10.1007/s10143-024-02578-8 (PMC11281945; doi:10.1007/s10143-024-02578-8)
Supplement: Supplementary file 1 — Supplementary Material 1 [file 10143_2024_2578_MOESM1_ESM.docx]

## Multiple Craniotomies in Patients with Brain Metastases: A Two-Center, Propensity Score-Matched Study

Luis Padevit (ORCID [0000-0003-2461-0404](https://orcid.org/0000-0003-2461-0404))^1^, Anna Maria Zeitlberger (ORCID 0000-0002-9837-5534)^2^, Nicolai Maldaner (ORCID 0000-0003-0284-1033)^1^, Johannes Sarnthein (ORCID 0000-0001-9141-381X)^1^, Oliver Bozinov (ORCID 0000-0002-4620-3992)^2^, Luca Regli (ORCID 0000-0003-4639-4474)^1^, Marian Christoph Neidert (ORCID 0000-0003-2828-4706)^2^, Carlo Serra (ORCID 0000-0002-7305-550X)^1^, Stefanos Voglis (ORCID 0000-0002-1514-1442)^1,*^

^1^Department of Neurosurgery, Clinical Neuroscience Center, University Hospital and University of Zurich, Zurich, Switzerland

^2^Department of Neurosurgery, Cantonal Hospital St. Gallen, St. Gallen, Switzerland

Running title: Patients with multiple craniotomies: A two-center propensity score-matched, retrospective cohort study

*Corresponding author:

Stefanos Voglis MD – [stefanos.voglis@usz.ch](mailto:stefanos.voglis@usz.ch)

ORCID: 0000-0002-1514-1442

Department of Neurosurgery, Clinical Neuroscience Center

University Hospital Zurich

Rämistrasse 100, 8091 Zurich, Switzerland

## SUPPLEMENTARY TABLES AND FIGURES

| **Grade** | **Definition** |
| --- | --- |
| 1 | Any deviation from the normal postoperative course without the need for pharmacological treatment or surgical, endoscopic, and radiological interventions. Allowed therapeutic regimens are drugs as antiemetics, antipyretics, analgetics, diuretics, electrolytes, and physiotherapy. This grade also includes wound infections opened at the bedside. |
| 2 | Requiring pharmacological treatment with drugs other than such allowed for grade I complications. Blood transfusions and total parenteral nutrition are also included. |
| 3 | Requiring surgical, endoscopic, or radiological intervention. |
| 3a | Intervention not under general anesthesia. |
| 3b | Intervention under general anesthesia. |
| 4 | Life-threatening complication requiring intensive care unit stay |
| 4a | Single-organ dysfunction (including dialysis). |
| 4b | Multiorgan dysfunction. |
| 5 | Death of a patient. |

**Online resource 1 Clavien-Dindo Grading System (CDG)**


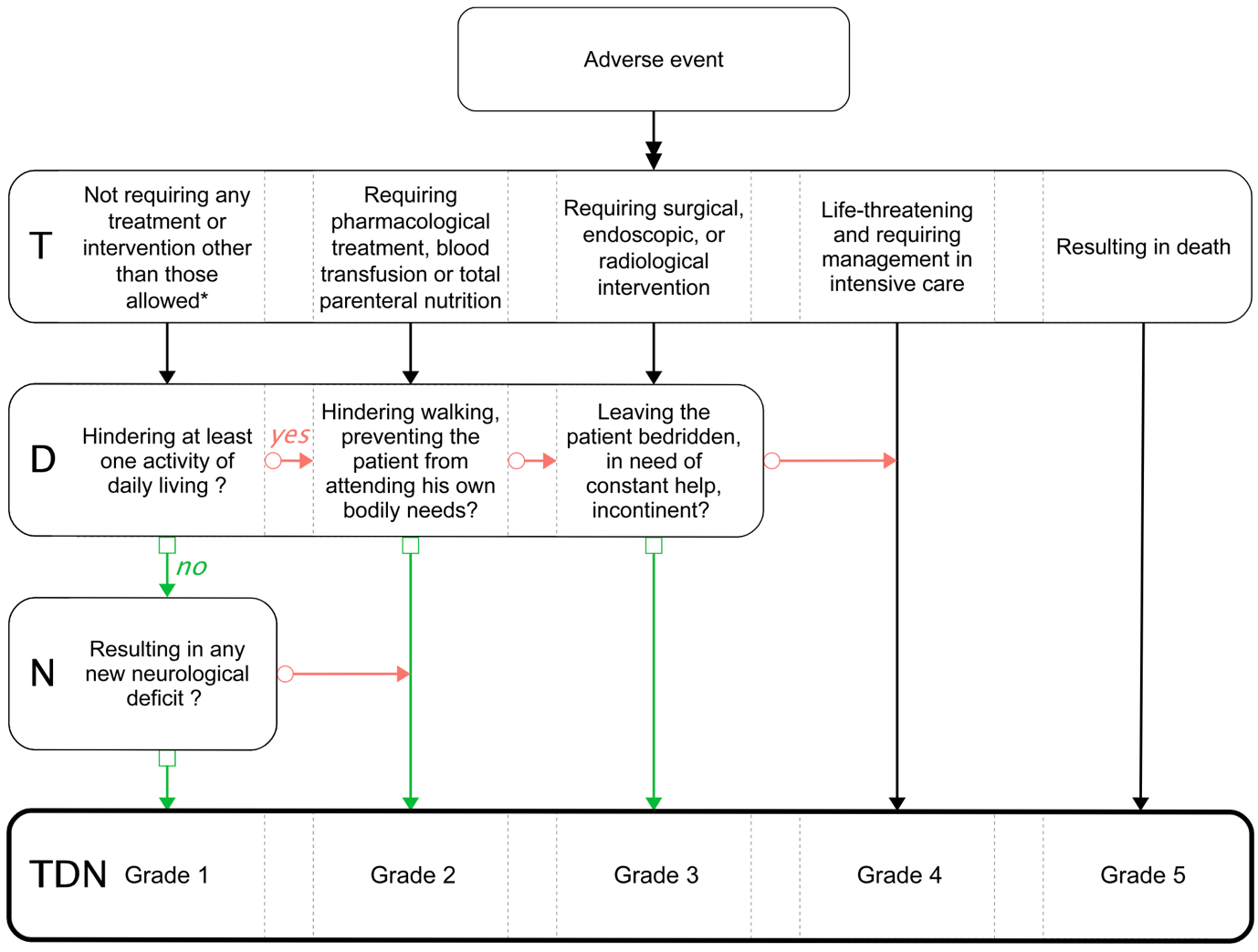


**Online resource 2 Therapy-Disability-Neurology Grade (TDN Grade)**

##
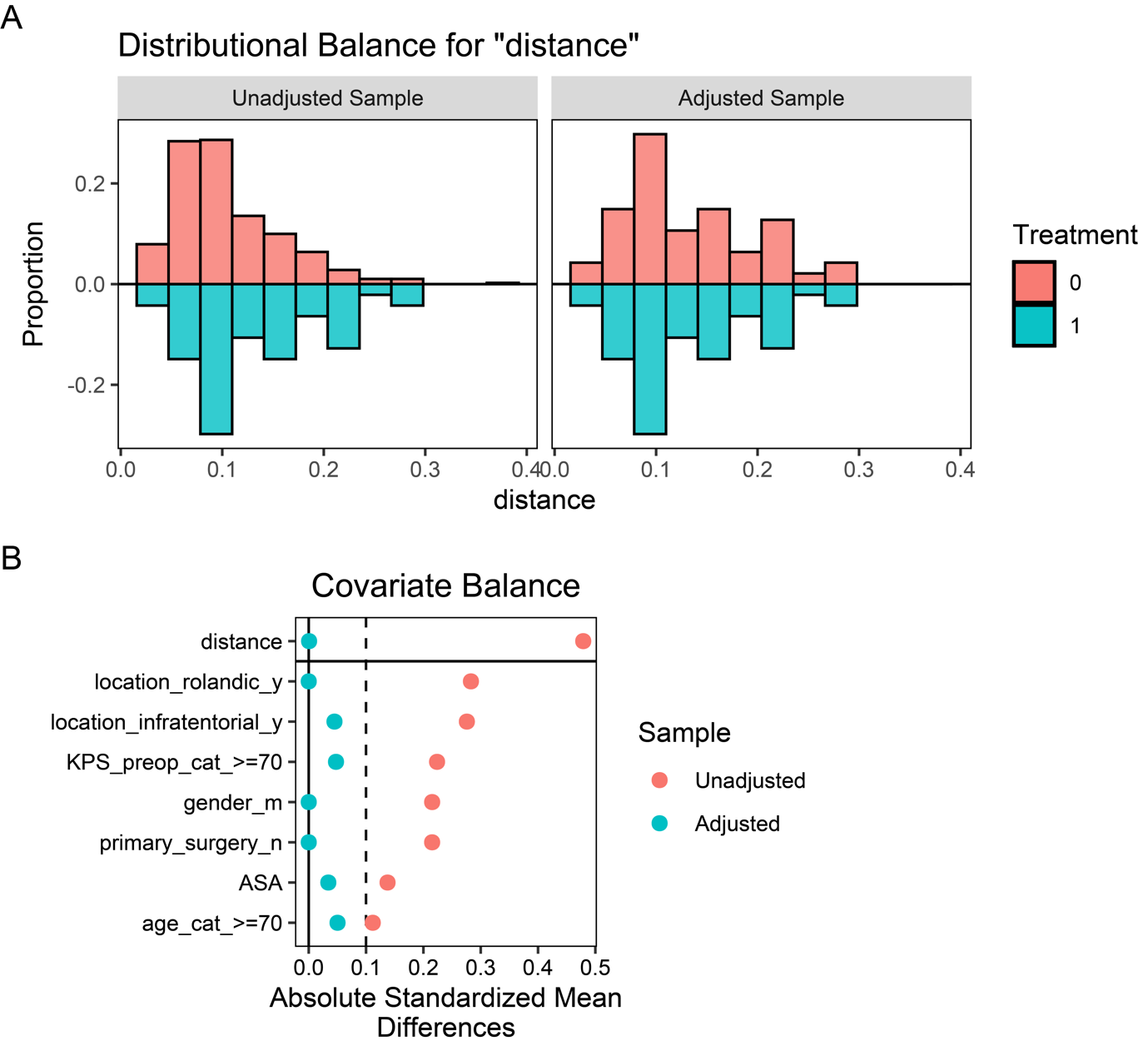


**Online resource 3 Matching results**

(A) Histogram indication the distributions of the distance measure before and after sample adjustment. (B) Love plot indicating covariate balances before and after sample adjustment. An absolute standardized mean difference threshold of < 0.1 after matching was considered as an appropriate balance


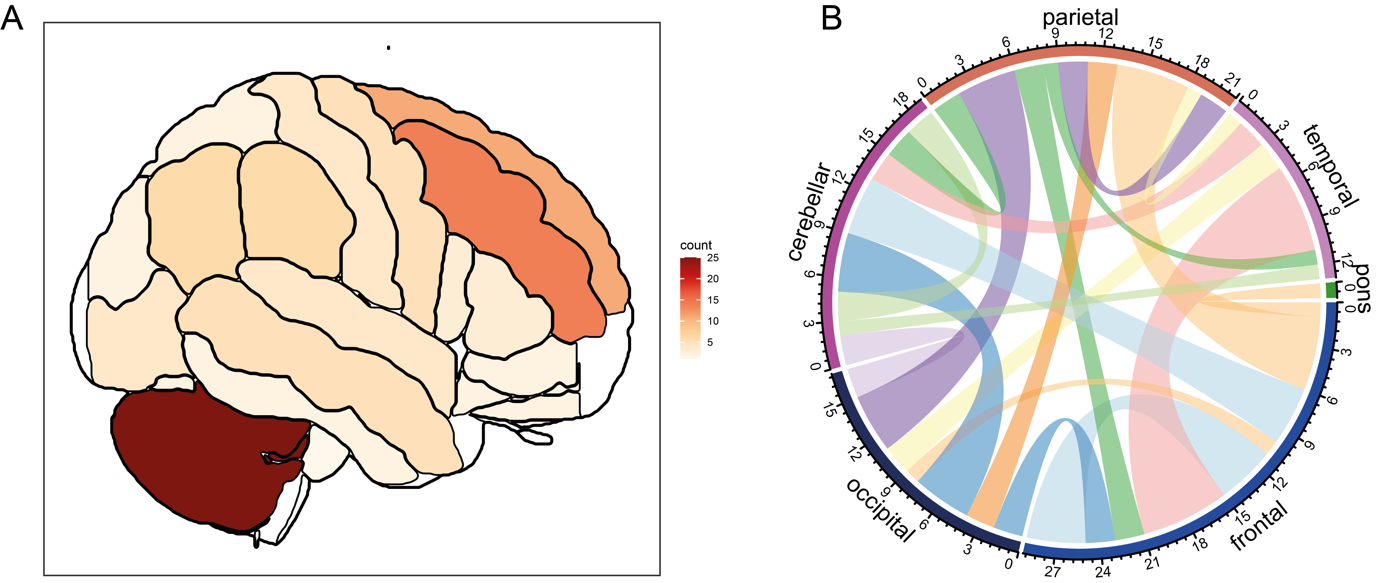


**Online resource 4 Anatomical BM location and chord diagram of multiple craniotomy locations**

(A) Anatomic location of the BM in the study cohort. (B) Chord diagram showing the corresponding lobule location of BM resected through multiple craniotomies

BM brain metastases
